# Impairment Arguments, Interests, and Circularity

**DOI:** 10.1093/jmp/jhae023

**Published:** 2024-05-13

**Authors:** Stephen Napier

**Affiliations:** Villanova University, Villanova, Pennsylvania, USA

**Keywords:** *abortion*, *circularity*, *harm*, *moral risk*, *time-relative interests*

## Abstract

A common justification for abortion rights is that the death of the fetus does not violate any of the fetus’s time-relative interests. The time-relative interest account (TRIA) of harm and wrongdoing tells us that a necessary condition for harming someone is that his or her time-relative interests are frustrated. Regarding the justification for abortion, this account falls prey to impairment arguments. Impairment arguments entertain cases of prenatal injury, such as the mother using illicit drugs that disable the child. The intuition is that the child who is born with such disabilities is harmed by the mother’s drug use. But it is unclear what time-relative interest is violated in cases of prenatal harm. Typical responses to impairment arguments point out that the abortion case is different because the child does not exist to experience such harms; but in prenatal injury + survival cases, the child does live to experience those harms. Thus, the TRIA justification for abortion is not impugned by impairment counter-examples. This article argues that this response to impairment arguments is viciously circular. The response must say that so long as you kill the child, no harm is done. But this assumes that killing itself is morally inconsequential and is not itself a case of harm. The response to impairment arguments, then, assumes the permissibility of abortion.

## I. INTRODUCTION

Suppose I believe that the Earth is flat. You point out that ships that sail away slowly pass out of sight as if they are going down. If correct, that visual presentation would be inconsistent with the Earth being flat. I reply that the electromagnetic field of the Earth distorts light waves to make objects emitting those waves look like they are going down. Flummoxed, you ask why I believe that such electromagnetic waves exist, let alone that they exert the visual effects I attribute to them. I respond that a flat Earth produces such electromagnetic waves (and I offer no independent reason for this claim). Frustrated, you point out that I am providing a circular justification. I am using the conclusion of an argument to deflect a claim against the premises of my very argument.

This article argues that a common justification for abortion rights must recapitulate a similarly circular epistemic structure. (It may be that pro-life justifications suffer the same structural errors, but they are not the subject of this article.) For the purpose of this essay, it is enough to show that one common justification for abortion rights is circular in ways structurally like my defense of the flat Earth thesis. (What is so wrong about such circular reasoning is explored in Section IV.). Specifically, the present article argues that an argument for abortion rights based on a time-relative interest account (TRIA) of harm is circular. The important development to note is that the present argument does not rely on views regarding personhood or bodily rights.

Section II describes the key concepts of the TRIA of harm or wrongdoing, and how TRIA justifies the permissibility of abortion. Section III explains and defends the impairment argument and demonstrates that TRIA must respond to it in a circular fashion. Section IV explains why the circularity is a serious problem for the TRIA justification of abortion. The basic pattern of my argument is as follows. Start the dialectic with a view on what counts as harming or wronging someone. This view purports to justify that killing preconscious human beings is permissible (I abbreviate this position as “permissible killing” or (pk)). There are cases, however, where it looks like preconscious human beings can be subjects of harming or wronging. To deflect such putative counter-examples, the defender of (pk) must provide a rejoinder. The bulk of my argument is to argue that this rejoinder is justified only if one assumes that (pk) is true. Section V concludes the article.

## II. THE TRIA AND THE JUSTIFICATION FOR ABORTION RIGHTS

Arguments for the permissibility of abortion are divided into two broad strategies: (a) the no-person strategy and (b) the person-but-lacks-feature × strategy.^1^ The former category of arguments argues that unborn human beings are not persons, and non-persons may be killed. Different arguments are presented for what counts as a human person, but for all of them, unborn human beings—especially at the embryonic or zygotic stage—are not persons (Tooley, 1972; Warren, 1973; 1992; McMahan, 2002). The latter category (II) of arguments typically grants that unborn human beings are persons in an ontological sense (Thomson, 1971; Boonin, 2002; Degrazia, 2005) they may grant the truth of the claim “my mother first felt *me* kicking when I was 18 weeks old.” But proponents of this strategy argue that unborn human beings lack some morally important feature, the lack of which makes it permissible to kill us, even if we are already born (Singer, 1979) or just about to be born (Degrazia, 2005). Two types of features have been proposed: interests (McMahan, 2002; DeGrazia, 2005) and bodily rights (Thomson, 1971; Boonin, 2002). This article focuses on arguments from interests, namely, the TRIA of harm or wrongdoing.

The TRIA is an explanation for why certain states of affairs (SOA) can be the object of rational egoistic concern. What matters to subject S is whatever in which S can take an interest, and this is a function of two factors. The first is the importance to S in realizing (or avoiding) an SOA, and the second is the degree of psychological unity between S now and when S realizes (or avoids) the SOA in the future. Having a time-relative interest requires meeting both conditions.

The extent to which one ought now to be egoistically concerned about that event is a function of two factors: first, the value, positive or negative, that the event would have at the time when it would occur, and second, the extent to which the prudential unity relations would hold between oneself now and oneself at the later time… (McMahan, 2002, 79; see also DeGrazia, 2005)

One must add a third necessary condition, namely, the capacity for having time-relative interests at all, which includes the capacity for consciousness, sentience, and memorial capacities. Psychological unity is a function of having the same brain, understanding “the same” as physical, functional, and organizational continuity, that is, the preservation of those brain areas associated with consciousness (McMahan, 2002). It is not necessary to remember everything I desired, say, ten years ago to be psychologically unified with myself ten years ago. All that is required, on McMahan’s view, is an overlapping psychological unity which is anchored in having the same functional brain.

TRIA proponents also think that what explains rational egoistic concern can also explain what counts as a harm. Hitting a tennis ball with my racket is not morally charged, hitting my cat with my racket is; the former does not have any interests, the latter does. More technically, if there is an SOA for which there are weak or nonexistent psychological unity relations between me now and the SOA in the future, I cannot be harmed by being deprived of that SOA. If a toddler cannot foresee, intend, or understand having chocolate cake for breakfast, not serving him the cake cannot matter to him. From his own perspective, he misses nothing by having Cheerios instead. Thus, if an SOA does not *matter* to me, I am not harmed by not experiencing it. The upshot of this is: if it is not me who will survive into the future, or the psychological unity relations are weak, as is the case between an early developing human being and an adult, survival cannot be an object of rational egoistic concern survival cannot matter to me.

The idea of “mattering” is used to describe a rational egoistic concern. But there is an informative objection reply that reveals a deeper understanding of TRIA relevant to the impairment arguments discussed below. If we interpret the question “What matters to subject S?” as asking what, from the first-person perspective, is understood by the agent herself as being an important SOA to realize, having a time-relative interest seems essential. If we interpret the question “What matters to S?” as asking what would redound to S’s benefit understood more objectively, having a time-relative interest is not essential. If so, then one might harm someone who is not conscious of the harm or cannot understand it as a harm. The reply is that it is hard to understand how S is harmed (or benefited) by SOA’s for which S cannot or will not ever experience. An unexperienced harm, as far as S is concerned, is unintelligible on the TRIA.

The TRIA of rational egoistic concern can function as an argument for the permissibility of abortion, and such an argument would not have to make assumptions about personhood. Grant that you and I are ontologically identical to the developing human being in utero; the TRIA claims that because the psychological unity relations are so weak or nonexistent, there is no harm to the fetus if she or he does not survive. She is like a child who is deprived of chocolate cake for breakfast. DeGrazia’s argument for the permissibility of abortion, for example, focuses on the moral relevance of interests according to which the preconscious child^2^ does not have the requisite interests to ground a claim that she or he can be harmed. The centerpiece of this justification is the idea of a time-relative interest which explains our intuitions that harm is parasitic on “the way in which one is psychologically ‘invested’ in, or connected with, one’s future” (DeGrazia, 2005, 286). The notion of interest that appears relevant is that one must be able to *take* an interest in continued living, which implies having an actual interest.^3^ But this “taking” is a degreed concept. DeGrazia states that “even if the presentient fetus has an interest in remaining alive, it would be too weak to ground a right to life” (2005, 288). It is too weak either because the fetus cannot form an interest, given that her or his cognitive and conative abilities are too incipient, or one’s memory capacities are too incipient to ground any *connectedness* between psychological states.

So, there is a triad of concepts; wrongdoing, harm, and interests that are related roughly as follows. Wrongdoing requires harming, and harming requires violating someone’s time-relative interests. Having an interest requires being conscious or having enough mental capacity to take an interest in something. Since preconscious human beings cannot take an interest in continued living, ending their lives cannot violate or frustrate any of their time-relative interests. Therefore, preconscious human beings cannot be harmed. If they cannot be harmed, killing them is permissible. Therefore, killing them is permissible.

## III. IMPAIRMENT ARGUMENTS

Impairment arguments are arguments against the TRIA of harm, according to which there are cases where a subject is clearly harmed by another agent’s actions, but, intuitively, there is no time-relative interest that is violated or frustrated. Specifically, impairment arguments focus on putative harms on preconscious human beings. The idea is that if such harms are possible, then the fact that one cannot violate a time-relative interest is still not sufficient to justify permissibly killing preconscious human beings. That is, if impairment arguments are successful, they justify the following claim:

(Impairment): If it is possible to harm preconscious human beings, then it is not necessarily the case that if there is no time-relative interest that is violated by such an action, then it is permissible to kill that preconscious human being.

The idea behind impairment arguments is that if harm is possible *sans* violating a time-relative interest, then killing that preconscious human being could count as harm.

The following are some putative counter-examples to motivate impairment arguments. Consider prenatal handicapping according to which an agent harms the preconscious child, for example, the mother abuses drugs voluntarily while pregnant with the child (DeGrazia, 2005; McMahan, 2002; see also Montague, 1989). The intuition is that the mother does something impermissible in harming the preconscious child. But if that child does not have or has very weak prudential concerns for the future, how can we explain the intuition that the mother’s action is wrong just on TRIA?

Ingmar Persson (1999) describes a case of neuron removal to illustrate the possibility of preconscious harm as well.

Consider a series of removals of neurons from the brains of preconscious fetuses… Imagine that this piece of surgery could be performed without killing the fetuses, but that it would cause them to be mentally retarded to some degree. If one removed just a few neurons, this would harm the fetus… If it would restrict its mental life and thus make it less capable of leading a worthwhile life. If one removed still more neurons, this would harm the fetus more were it to make its future life even less worthwhile… Suppose, finally, that so much of the fetus’s cerebrum is removed that it never acquires any consciousness at all… Then, according to [a view that harm requires consciousness], this would not harm the fetus. (Persson, 1999, 301)

If having future experiences is a necessary condition for being harmed, then it looks as if the preconscious child is harmed by neuronal removal *except when* that removal undercuts the possibility of developing consciousness at all. However, Persson observes correctly that this ultimate manipulation inflicts a “*greater* harm on the fetus than the penultimate manipulation did…” (1999, 302).^4^

Last, consider a case of preterm injury plus restoration. Suppose a preterm mother suffers domestic abuse and as a result, the preconscious child is smitten in the womb such that he suffers severe brain damage. Suppose that there exists a fetal brain surgery to correct the damage and in this case is completely restorative. In this case, the child is harmed *by the battery*, for if not, there is no sense in which we can say that the surgery was *restorative* or even that the surgery is *needed*. Those descriptors make little sense without supposing that an injury has occurred. This case suggests that harms can occur to an agent without that agent experiencing the effects of those harms.

### Response to Impairment Arguments

DeGrazia correctly notes that such actions are wrong because the preconscious child has a “present time relative interest in being healthy but also many future time relative interests in being healthy…” (2005, 289). DeGrazia also correctly recognizes the problem saying, “… Since the presentient fetus [in the mother-drug case] is psychologically cut off from the child he will become, his time relative interest in later being healthy is extremely weak, just as his time relative interest in staying alive is extremely weak. How, then, to explain the judgment that the woman’s behavior is seriously objectionable…?” (2005, 289).

It would seem to follow that the preconscious child who is killed by an abortion would also have a present time-relative interest in being healthy. If the drug-abused child is harmed and harming requires having interests, the drug-abused child must have interests that are violated. If the drug-abused child can have such an interest, so may the child who is killed by an abortion, since we may easily assume that they are developmentally similar vis à vis having interests. And so, it would follow that killing the child involves a significant harm as well. So, if the preconscious child is harmed by prenatal handicapping, the preconscious child who is killed is also harmed. Both are wronged.

All the authors who consider impairment arguments agree that in cases where the child *survives* prenatal harm, the child is wronged. There is only one option open to the TRIA justification for permissible killing (pk). The handicapped child is harmed but the aborted child is not because of further specifications on what counts as morally relevant interests. If we stick to what proponents of the TRIA say, those specifications take the following two forms.

(1) Experience condition. In nonabortion harm cases, the child survives into adulthood and experiences the effects of handicapping (e.g., the mother’s drug habit or neuronal removal). The drug case is immoral because the child *will* exist into the future and *experience* the harms that were originally caused by the mother’s drug habit. It is only *at the point of experiencing* the harms that one can say the person is harmed. DeGrazia intimates this option when he comments on the drug-abused child: “*Because the woman decided against abortion*, her fetus has not only a present time-relative interest^5^ but also many future time relative interests in being healthy…” (2005, 289). And for the aborted child, he states that “… we count only the fetus’s present time relative interest to live, because an aborted fetus *will never have time relative interests* while psychologically deeply unified…” (2005, 289, emphasis added). When explaining the TRIA, DeGrazia says, “[t]he essential idea is that prudential evaluation of a possible future… should take into account both the value of that future to one *as one experiences it* and how psychologically invested one now is in that future” (2005, 191, emphasis mine). Likewise, McMahan observes that “abortion affects its victim only when the victim is a fetus with weak time relative interests, prenatal harm affects its victim *later*…” (2002, 282, emphasis mine). McMahan thinks that the moral relevance of this distinction is simply that the fetus in the abortion scenario will not have any future time-relative interests, because she or he is killed;^6^ but if that abortion is not performed, the fetus will have time-relative interests. And if the aborted child cannot have any future time-relative interests, she or he cannot be harmed. Likewise, Blackshaw, following McMahan (2006) says that “killing the fetus entails that there is *no* future individual with interests to be damaged. Consequently, the *reason* why [inducing fetal alcohol syndrome] to a fetus is immoral does not apply to the act of killing the fetus” (Blackshaw, 2019, 724). So, for S to be harmed, S must experience the harm. Since the aborted child does not experience anything, neither actual nor future interests are frustrated, and consequently, no harm either.^7^
(2) Content specification. The second option is to specify the content of the time-relative interest. DeGrazia suggests this option when he supposes that in the abortion scenario “we count only…the present time-relative interest *to live*…” (2005, 289, emphasis added). The idea seems to be this: both the aborted and the drug-abused child cannot form an interest in continued living, since that requires having a self-concept, and projecting one’s self as existing into the future. With no interest, no harm. But the drug-abused child has an interest in being *healthy*, albeit a weak one.

Content specification holds that the handicapped child is harmed *at the time* of the drug abuse or neuron removal but that the aborted child does not form an interest to live, and so *that* interest cannot be violated. And the Experience condition holds that the handicapped child is harmed *only when* the effects of the drug abuse or neuron removal can be experienced. The Experience condition tells us that neither the aborted nor the prenatal handicapped child is harmed at the time that the putative harms are caused. If the handicapped child were to die of sudden infant death syndrome prior to experiencing the harms of drug abuse, there still is no wrongdoing, given the Experience condition.

Of course, the normal default attribution of wrongdoing is that it occurs when the injury occurs. In the battery case, for instance, the child was wronged at the time of battery. Consider a case of unethical gene therapy research according to which the effects are not realized for another few years. Intuitively, researchers performed a wrong act *at the time* they did, say, a risky gene therapy infusion, but the experience of the harms is displaced in time. The proponent of the Experience condition, however, places emphasis on the fact that *as far as S is concerned*, unexperienced harms do not matter to her. And if they cannot matter to S, S cannot be wronged.

### Why the Response is Circular

I can now argue that the TRIA justification for (pk) is circular. Impairment arguments function as a defeater to TRIA’s justification for (pk). Prior to considering impairment arguments, the TRIA justification has it that if S lacks the ability to have a time-relative interest in continued living, S cannot be harmed. Impairment arguments suggest that harms can occur even in the absence of such time-relative interests.

Consider the Experience condition first. This condition functions as a defeater *deflector* to the impairment argument, thereby preserving TRIA’s justification for (pk). The upshot of that condition is that we have two classes of cases: prenatal harm plus survival, and prenatal killing (i.e., abortion). The only apparent difference between the two classes of cases is that the child is killed in the abortion case, but not in the survival case. Grounding a moral asymmetry between these cases must say that *killing itself* is the difference maker, such that the child in the abortion case is not harmed, but in survival cases the child is harmed. So long as one kills preconscious human beings, it does not matter how many neurons are removed, or how many parts of the brain are damaged from battery or illicit drug use.^8^

But this claim *assumes* that killing is not an instance of harming. The abortion doctor does not do anything wrong in the abortion case because the child is neither conscious (of the harms) nor will she ever be. If killing itself is the difference maker, which entails that the child is not wronged if she or he is killed, then deflecting impairment arguments must assume that killing is permissible. So, to deflect impairment arguments, the TRIA justification for (pk) must already assume that killing preconscious human beings is morally permissible. The so-long-as-one-kills response amounts to saying that those counter-examples pose no threat *in the abortion case* because *killing preconscious human beings is morally permissible*. The background assumption that adjudicates whether various types of prenatal harm cases count against the TRIA is the very claim that needs argument, namely, (pk). Therefore, (pk) is presupposed to deflect a defeater to a premise in the argument for (pk). Such an argument is circular in that the claim that needs defending, that is, (pk), is being presumed.

Consider now the Content Condition. If what makes it immoral to harm the child in the first case is that the child has a present time-relative interest in being *healthy*, then the same interest is sufficient to say that *killing* the child counts as a harm insofar as not existing is incongruent with being healthy. The issue here is not that existing is merely a necessary condition for being healthy. My claim is that if S were to have an interest in being healthy, that interest entails existing, regardless of whether S forms the latter interest explicitly. Suppose a vapid teenager forms an interest in being healthy but never explicitly makes the inference that she must exist as well. She still enjoys a right not to be killed. If it is an *ability* in taking an interest in continued living that matters, the conceptual content of “being healthy” is no more complicated to form than forming an interest in living.

Furthermore, why “count only” the interest that happens to preserve the belief that abortion is permissible? If the “count only” clause is there *so that* permissible killing is preserved from counter-example, then we have an instance of circular justification again. If it is based on independent reasons, what could they be? DeGrazia does not say.

To summarize, the only reason for intercalating deflectors to counter-examples of preconscious wronging is to make consistent one’s commitment to (a) an interest account of harm/wrongdoing, (b) one’s belief that some cases of prenatal wronging can occur, and (c) one’s belief that abortion is permissible. When faced with a putative example of preconscious harm, one can either accept that harms and interests are not coextensive (in which case one loses a justification for abortion strictly on TRIA); or one can argue that contrary to appearances, they are coextensive. Arguing that they are coextensive contrary to appearances requires (1) or (2) as further specifications. The Experience condition presupposes (pk), and therefore the TRIA justification for (pk) is circular. Content specification is independently implausible.

Before transitioning to the last section, it is important to note that my argument for vicious circularity does not require surveying all the intuitions that serve to motivate the TRIA. My argument is simply the claim that in deflecting a defeater, namely, endorsing the Experience condition, the TRIA justification for (pk) must presuppose (pk) itself. This is a structural defect; it is an epistemically circular argument.

## IV. WHY IS THIS CIRCULARITY A PROBLEM FOR THE TRIA’S JUSTIFICATION FOR (pk)?

Even if my argument is correct that the TRIA argument for (pk) is circular, one might complain, asking cynically, “So what if they are circular?” Arguments for the reliability of one’s cognitive faculties are circular (Alston, 1993), but we do not endorse global skepticism over that fact. A related objection is that the argument from TRIA to (pk) is, at least, a self-contained coherent system. Though it cannot prove alternatives false, it is a coherent system of mutually supporting beliefs. Coherence has typically not been taken as a reason for skepticism.^9^

The first reply that comes to mind is that such a response is hypocritical. For instance, Bigelow and Pargetter say in their critique of pro-life arguments the following, “[b]ut now the issue is to justify, in a manner which *does not beg-the-question*, the claim that the conceptus does have potential in this sense to be a person…” (1988, 178–9, emphasis mine). Boonin also endorses similar epistemic standards in his critique of pro-life arguments. “I’ve been asking whether there is a reason that can be given for making species membership morally relevant, a reason that could be grounded in some other belief or set of beliefs *that critics and defenders of abortion both are likely to share*” (Boonin, 2002, 27, emphasis mine). Further on, Boonin remarks in response to a different pro-life argument that “while this claim may turn out to be true, it simply is not plainly true. And *in the context of the debate about abortion*… *it cannot reasonably be simply assumed to be true*…” (Boonin, 2002, 53, emphasis mine). And on the next page, he reiterates the epistemic standard he is assuming, stating it as follows. “I am concerned in this book to examine those arguments with which a critic of abortion *can attempt to convince those not already committed to the thesis that abortion is morally impermissible*” (Boonin, 2002, 54, emphasis mine). Since pro-life persons are not already committed to the permissibility of killing preborn human beings, etc., the TRIA justification for abortion turns out to be a bad argument on Boonin’s and Bigelow & Pargetter’s standards.

Claims of hypocrisy are rhetorically advantageous, but they are not where I wish to put emphasis, since they bear an uncomfortable propinquity with *tu quoque* arguments. The more important aspect of such observations, though, is that they reveal an important agreement on norms of discourse. Norms of discourse, particularly in moral philosophy, require giving and responding to reasons. This is especially true in settings of peer disagreement according to which there exist plausible challenges to one’s conclusions (Beckwith, 2007; Lee, 2010). In such a setting, proponents of such conclusions are understood to *give* reasons and *to justify* their position. These norms of discourse are endorsed by my interlocutors, per the quotations above.

### Do Circular Arguments Succeed in Meeting These Norms?

Those who argue that circularity is benign typically do so on two grounds. The first is if there are independent reasons for the claim that is the conclusion of a circular argument (Walton, 1985). The second ground is if there is no other way to argue for p. This ground occurs mainly in discussions on proving the reliability of one’s cognitive faculties. The circularity is clear: any argument for the reliability of one’s cognitive faculty F must use that very faculty as an argument in support of its own reliability. The conclusion of such an argument, namely, F is reliable, must be assumed in defending the premises such as, F produced belief B *and B is true*. Thus, those who think that circularity is benign start by observing that one can have noninferentially justified beliefs (Bergmann, 2004). A good candidate for a noninferentially justified belief is that my faculties are reliable. Why *must* one endorse such a claim? The reason is that if the skeptic thinks that arguments for the reliability of one’s faculties are circular and circularity is epistemically bad, then all of our faculties are called into question, including the faculty that generates the skeptical argument itself. The skeptic, then, risks self-defeat.

In the present discussion, neither ground is applicable. Any reason for thinking that (pk) is true independent of the TRIA would no longer be an argument based on the TRIA. And denying either (pk) or TRIA does not lead to self-defeat. The reason circularity infects proofs for the reliability of one’s *faculties* is because we cannot but think, perceive, or generate proofs without those faculties. Believing (pk) or TRIA is not a precondition for thinking the way believing in the reliability of one’s own faculties is a precondition for thinking.

So, features that would make circularity benign do not apply to the circularity infecting the TRIA justification for (pk). Why think that the circularity is vicious? Most commentators on epistemic circularity agree that what makes epistemically circular arguments vicious is that they fail to justify their conclusions in actual dialectical encounters. Walton explains as follows,

[T]here may be one special kind of context where a circular argument can be seen to violate a reasonable procedural requirement of good dialogue. This context occurs where it is acknowledged by both players that each must prove or argue from premises that the other accepts as *more plausible* than the conclusion each prover is supposed to establish. (1985, 271)

Walton accepts that circular arguments might be formally valid, but in these special contexts, they do not provide a “‘useful inference’ if the premises are as doubtful as the conclusion” (Walton 1985, 271–2). The abortion debate is such a context in which such a procedural requirement needs to be met.

Cling (2003) extracts similar motifs in his treatment of circularity, focusing specifically on the dialectical role that argumentation plays. In his terminology, justification-affording arguments for a claim C give one’s target audience justification for believing in C. Within the class of justification-affording arguments there are those that are justification-creating, according to which such arguments “create justification for belief in their conclusions” (Cling, 2003, 281) specific to one’s target audience. Additionally, there are those that are justification-enhancing, according to which such arguments “enhance the justification that their target audiences already have for believing their conclusions” (Cling, 2003, 281). Epistemically circular arguments cannot be justification-creating, however. “For an argument P therefore C is justification-creating for audience S only if it is such that *if S were not justified* in believing that C, then S *could* come to be justified in believing that C by reasoning through P to C” (Cling, 2003, 287, emphasis mine). Epistemically circular arguments (self-supporting arguments on Cling’s terminology), however, cannot create justification. “For in such a case S’s justification for believing if P, then C depends upon S’s justifiably believing that C” (Cling, 2003, 287). So, S would have to justifiably believe C already, in which case circular arguments are not justification-creating. S could not *acquire* justification for C if C is already presupposed. Circular arguments do not give one reason to acquire new beliefs or to change the ones they already have.

Sgaravatti argues that circular arguments are bad for very similar reasons, namely, they are dialectically inert, they give one’s interlocutor no reason for acquiring new beliefs or changing the ones they already have. Sgaravatti explains that through circular arguments

… you cannot *acquire* a justified belief in their conclusion, assuming that you cannot acquire a justified belief by inferring from unjustified premises… whenever the conclusion is actually doubted or is believed without justification, we cannot improve on that situation by making use of the [circular] argument. (2013, 771)

Alston too voices these same concerns about the force or usefulness of circular arguments.

The reason why epistemic circularity is important in this context is that arguments that are infected with it would seem to have *no force*. If we have to assume [belief in the reliability of perception (RP)] in order to be entitled to premises for an argument for it, how can the argument provide support for RP? If our assumption of RP is warranted before we give the argument, how does the argument *add* to the [positive epistemic status] of RP? (Alston, 2005, 202, emphasis mine)

Finally, Bergmann notes that circular arguments can be benign when the premises of such arguments are justified noninferentially, viz. they are first principles. He explains how they can be bad as follows. “The sort of belief that can get infected with epistemic circularity is a belief that one’s belief source, X, is trustworthy. A context in which epistemic circularity is a bad thing is one in which the *subject begins by doubting or being unsure* of X’s trustworthiness” (Bergmann, 2004, 717, emphasis mine).

The common theme here is that circular arguments are bad when a conclusion or premise in that argument is questioned, doubted, or directed to an audience that does not already believe the premises or conclusion. In such contexts, circular arguments are inert or dialectically useless. They are not justification-creating in Cling’s terminology; nor do they justify their conclusions in Alston’s terminology.

We can now see why the circularity infecting the TRIA justification for (pk) is vicious. The commentators above agree on one aspect of circular arguments, namely, they are bad in dialectical contexts in which the proponent of the argument needs to prove or justify to a target audience his or her claim. It is precisely this *activity* of *justifying* a claim that circular arguments are unable to do. In the abortion context, what needs justifying is that it is permissible to kill a developing human being who is ontologically identical to you and me (DeGrazia, 2005), or a person as understood in Thomson’s (1971) argument, and McMahan’s (2002) argument (for late-term abortions). The proponent of such a position is saddled with the epistemic project of creating justification for that claim. I take it as uncontroversial that the discourse on abortion requires giving reasons and justifying one’s beliefs. Therefore, offering justification-creating arguments is the goalpost.

The reason why this is the goalpost is because my interlocutors agree with such norms. Even if they did not, however, there are independent reasons for thinking that providing justification-creating arguments is required.

The first reason is that the costs in being wrong (for either (pk) or not(pk)) are non-negligible (Moller, 2011; Napier, 2020). The cost in being wrong in believing (pk) and acting on it involves the unjust killing of an innocent person, or a human being ontologically identical to you and me. Such an action requires providing a justification-creating argument that addresses peer challenges from proponents of ~(pk). The belief that “abortion is permissible” has a high cost of being wrong if acted on. Beliefs with a high cost in being wrong require justification-creating arguments before permissibly acting on them mere coherence is not enough. This is especially true if the cost of being wrong is borne by someone else; in this case, the unborn human being. Circular arguments are not justification-creating arguments, per the reflections above. The TRIA justification for (pk) is a circular argument, per Section III. It follows that the TRIA justification for the permissibility of abortion is not a justification-creating argument. Therefore, the belief that abortion is permissible based on the TRIA justification may not be acted on.

Second, there exists *peer* disagreement about the merits of (pk). In the settings of peer disagreement, it is not enough to rely on justification-enhancing arguments (Christensen, 2007). Standards of premise acceptability typically require meeting challenges from one’s opponent. Freeman explains the problem of accepting premises simply because they form a coherent whole. “Of course, one condition making a premise acceptable would be its being adequately defended by cogent argument” (Freeman, 2005, 19). This is not a satisfactory condition on Freeman’s view, since

[i]f the defending argument is cogent, *its* premises must be acceptable and adequately connected to justify accepting the conclusion. Barring circularity, we must eventually come to basic premises, the starting points of reasoning. Under what circumstances, if any, are *these* premises acceptable without any further justificatory argument? (2005, 19)

Acceptable premises, on Freeman’s view, are those premises that can be presumed, and presumption is defined with reference to what a challenger must concede, not to mere coherence. In the setting of peer disagreement around (pk), (pk) is not an acceptable premise, and some find it positively outrageous. Therefore, it requires further support. That premise cannot function as a reason to rebut the impairment objection.

To summarize, the reasons why the TRIA argument for (pk) is viciously circular is that settling for mere coherence (a) violates norms of discourse that my interlocutors agree with, (b) Mere coherence fails to offset the cost in being wrong about acting on (pk), and (c) it fails to satisfy basic conditions of premise acceptability in the setting of peer disagreement.

## V. CONCLUSION

The problem I highlight in this article is that viciously circular arguments fail to justify their conclusions. So, if the TRIA justification for (pk) is viciously circular, it fails to justify (pk). I do not argue that the balance of relative plausibility between the TRIA and other axiological theories is on par. In this regard, I do not consider “*tu quoque*” arguments, but even so, I find the conclusion that the TRIA is merely coherent and on par with alternatives still a significant achievement.^10^ If the TRIA is on par with alternative viewpoints that justify ~(pk), given the cost of being wrong about acting on (pk), namely, one would intentionally kill someone who has a fundamental right not to be killed, one should not act on (pk). Arguably, the moral cost in being wrong about ~(pk) and acting on it is a violation of one’s liberty interest (Boonin, 2002). But all parties agree that killing a human person is worse than violating someone’s liberty interest—no one justifies killing children simply because the mother and father must alter their behavior and choices to care for the born child. Even if they really want to do other things with their time, caring for their born children is still obligatory. Failures to care, no matter how strong the liberty interest might be, are viewed as child abuse. So, in the setting of asymmetrical costs in being wrong about (pk), arguing for parity^11^ still delivers the judgment that abortions should not be performed if the putative justification has anything to do with the TRIA.

As I see it, the only option to controvert my argument is to explain why circular arguments can still function as good justifications for controversial moral claims. Of course, another option is to abandon the TRIA justification for (pk).

## Footnotes

1 The term “person” on this second strategy is not understood in any dualistic sense, it is not understood as differentiating between human organism and person. The term “person” on this second strategy refers to you and me at every point in our existence, which includes before birth. Thomson, for example, says, “I am inclined to think also that we shall probably have to agree that the fetus has already become a human person well before birth” (1971, 47). And further on she says, “I propose, then, that we grant that the fetus is a person from the moment of conception” (Thomson, 1971, 48). Famously, the Violinist analogy is meant to show that even a person does not have the right to the use of someone else’s body, viz., some persons lack a morally relevant feature that renders abortion permissible.

2 I use the term “preconscious child” because DeGrazia grants our numerical identity from the time after twinning is possible and onward. McMahan too thinks that late-term abortions are permissible even after the onset of consciousness a criterion for personal identity on his embodied mind view. McMahan’s argument for the permissibility of late-term abortion is that the prudential unity relations are too weak. Both think that the absence of a rational egoistic concern for the future is sufficient to say that the agent cannot be harmed. As such, the TRIA justification for abortion rights is meant to prescind from metaphysical questions of personhood.

3 I do not argue that TRIA is false in this article. But there are plausible arguments to this effect; see Kaposy (2007), Tollefsen (2010), Holtug (2011), and Nichols (2012).

4 See Hendricks (2019a, 2019b) and Blackshaw (2019) for important discussions on harms that come in progressive stages.

5 In saying this, DeGrazia seems to grant that the preconscious child at the time she is still preconscious has a present time-relative interest. Taking an interest seems to require having desires or at least some psychological capacity. So, it is implausible to suggest that a preconscious human being can also take an interest. Granted, he does say that the present interest is weak. But weakness on the TRIA is a function of psychological *connectedness* to one’s future (or past) psychological states which requires functional memory (McMahan, 2002). One could say that what matters here is having an objective interest, for example, a blade of grass wants to grow but is not conscious and does not technically take an interest in growing. But the moral relevance of setting back an objective interest is questionable. It is not immoral for me to mow my lawn, even though I am setting back the objective interests of my grass wanting to grow. Resorting to objective interests, then, would not explain why we think that the drug-abused child is wronged *at the time of the injury*. Given (1), wrongdoing seems to occur later.

6 Gillham echoes similar sentiments saying that, “[s]ince fetuses that will not be born do not have futures full of experiences, activities, projects and enjoyments to render their lives valuable, fetuses have no [future like ours]. Therefore… aborting them does not deprive them of an [future like ours]” (2021, e43).

7 The reason for describing this as the *experience* condition and not, say, as the future *interest* condition is because some forms of harming can render the formation of interests in the future rather difficult or impossible, as with progressive neuron removal. But we still think that if such a being lives to experience those harms, that is enough to ground claims of moral wrongdoing. If I were to focus too narrowly on interests being violated specifically, it would be too easy to generate counter-examples to TRIA since it is not hard to imagine that the child suffering from fetal alcohol syndrome fails to *ever* form an interest in continued living or in being healthy.

8 One reviewer noted that an implication of the TRIA at this point is that in cases in which a child survives an abortion but with serious injuries, one is *obligated* to kill the child to prevent the child from experiencing the effects of those injuries. The TRIA paired with the Experience Condition delivers this rather counter-intuitive consequence.

9 I thank an anonymous reviewer for pressing this objection.

10 I thank an anonymous referee for entertaining this possibility.

11
Boonin (2002) argues against cost-based arguments and, for the most part, I agree with his criticisms. But his criticisms do not consider parity in the relative *justifications* for (pk) and ~(pk)—witness his examples of cutting grass or one’s hair as involving mass murder. If the relative epistemic weight for (pk) and ~(pk) are on par, and the costs in being wrong are asymmetrical, one should avoid doing the action that is more costly.
